# Temperature and heat in informal settlements in Nairobi

**DOI:** 10.1371/journal.pone.0187300

**Published:** 2017-11-06

**Authors:** Anna A. Scott, Herbert Misiani, Jerrim Okoth, Asha Jordan, Julia Gohlke, Gilbert Ouma, Julie Arrighi, Ben F. Zaitchik, Eddie Jjemba, Safia Verjee, Darryn W. Waugh

**Affiliations:** 1 Department of Earth and Planetary Science, Johns Hopkins University, Baltimore, Maryland, United States of America; 2 IGAD Climate Prediction and Applications Centre (ICPAC), Intergovernmental Authority on Development (IGAD), Nairobi, Kenya; 3 Department of Meteorology, University of Nairobi, Nairobi, Kenya; 4 Department of Population Health Sciences, Virginia-Maryland College of Veterinary Medicine, Virginia Polytechnic Institute and State University, Blacksburg, Virginia, United States of America; 5 Red Cross Red Crescent Climate Centre, The Hague, Netherlands; 6 American Red Cross, Washington, D.C., United States of America; 7 Kenya Red Cross Society, Nairobi, Kenya; Universidade de Vigo, SPAIN

## Abstract

Nairobi, Kenya exhibits a wide variety of micro-climates and heterogeneous surfaces. Paved roads and high-rise buildings interspersed with low vegetation typify the central business district, while large neighborhoods of informal settlements or “slums” are characterized by dense, tin housing, little vegetation, and limited access to public utilities and services. To investigate how heat varies within Nairobi, we deployed a high density observation network in 2015/2016 to examine summertime temperature and humidity. We show how temperature, humidity and heat index differ in several informal settlements, including in Kibera, the largest slum neighborhood in Africa, and find that temperature and a thermal comfort index known colloquially as the heat index regularly exceed measurements at the Dagoretti observation station by several degrees Celsius. These temperatures are within the range of temperatures previously associated with mortality increases of several percent in youth and elderly populations in informal settlements. We relate these changes to surface properties such as satellite-derived albedo, vegetation indices, and elevation.

## Introduction

In a changing climate, heat and heat exposure are growing concerns. Currently, extreme temperature is one of the deadliest forms of climate hazard worldwide [1]. This burden is projected to increase as the climate warms, but will be unequally distributed, with heat exposure in some African regions, including East Africa, projected to increase by two orders of magnitude relative to that in Europe [2]. This presents a challenge to urban planners, public health officials and the disaster management community, since resource-poor countries may be ill-equipped to handle these challenges.

Heat exposure also has the potential to be exacerbated by increasing urbanization, which cause city temperatures to be hotter than the surrounding rural temperatures by several degrees [3]. These urban-rural thermal differences, often referred to as the urban heat island effect, have several causes: increased surface area from added buildings, increased heat retention from man-made materials, and decreased evapo-transpiration from clearing plants and vegetated surfaces [4]. However, urban areas are neither monolithic nor homogeneous. Different micro-climates cause temperature readings to differ by up to several degrees [5], meaning that a resident’s heat exposure may vary by or even within neighborhood. It may also mean that some areas need intervention when other areas do not. Understanding the existence of micro-climates within the urban setting can have important implications for the disaster management and public health spheres by creating opportunities for more targeted interventions to reduce the effects of heat exposure now and in a warmer climate.

Nairobi, Kenya exhibits a wide variety of heterogeneous surfaces. The central business district is characterized by paved roads, wide sidewalks, and high-rise buildings interspersed with low vegetation, while large neighborhoods of informal settlements or ‘slums’ are characterized by dense metal housing, little vegetation, and limited access to public utilities and government services. This and other informal settlements contain low, dense housing types built from galvanized iron sheets, wood and mud with inadequate access to basic services such as clean water. In 2003, the Government of Kenya and UN Habitat signed a memorandum of understanding with an objective of upgrading slums in Nairobi, but a substantial number of residents still live in informal settlements. Population counts in informal settlements are difficult to estimate precisely, but it is estimated that anywhere from one third to 60% of Nairobi’s 3.1 million residents live in informal settlements [6, 7], the largest of which is Kibera. The population residing in these settlements are potentially highly vulnerable to heat exposure due to lack of information on heat wave occurrence and risk, inadequate access to routine health services, limited access to potable water, limited household ventilation and lack of access to cooling centers.

**Fig 1:**
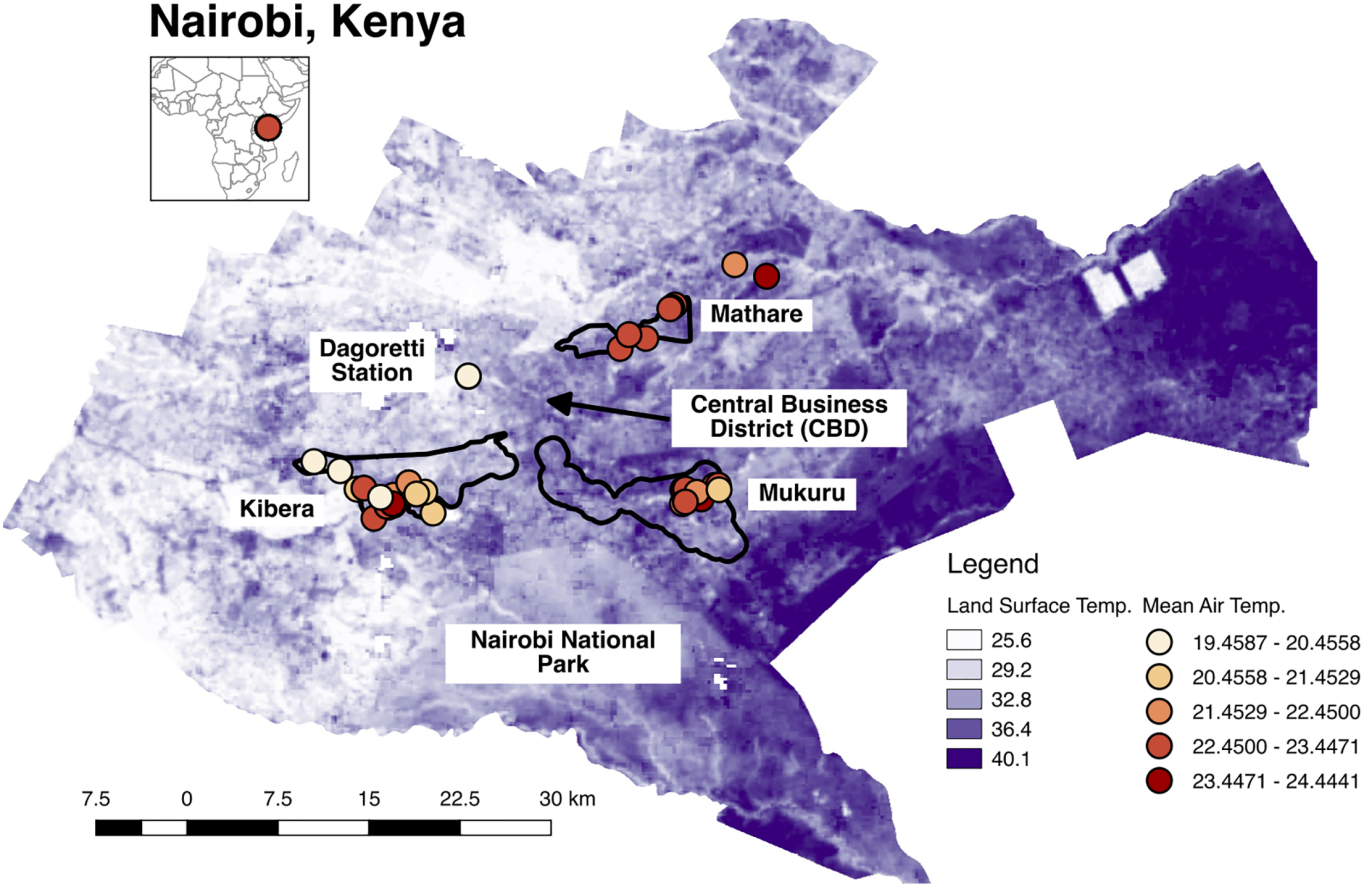
Map. Locations of iButton heat monitors in Nairobi, Kenya. Red colors represents the mean daily temperatures at each monitor; purple colors represent the land surface temperature calculated from Landsat8. Black outlines denote the Mathare, Mukuru and Kibra constituencies, the latter of which contains the Kibera informal settlement neighborhood.

Previous Nairobi field studies have found evidence of warmer urban temperatures [8], particularly for minimum daily temperature, and satellite thermal imagery shows that land surface temperature is warmer in the city and in informal settlements (Fig 1) than in some rural locations. An epidemiology study also found that heat is related to increased rates of mortality and morbidity in Nairobi’s informal settlements [9]. However, no study of temperature and thermal comfort in informal settlements has ever been conducted in Nairobi or other cities in Kenya (of which the authors are aware).

In Nairobi, the main observation station (Dagoretti) is located at the Kenya Meteorological Department Headquarters, just a kilometer away from the informal settlement of Kibera, but characterized by very different land cover type and architecture. In this paper, we ask if informal settlements are hotter than the central monitoring station, and if so, why, by investigating temperature, humidity and the thermal comfort or heat index in several informal settlements in Nairobi, Kenya. This question has important implications for heat exposure for residents of informal settlements in Nairobi and beyond.

## Methods

### Observations

**Fig 2:**
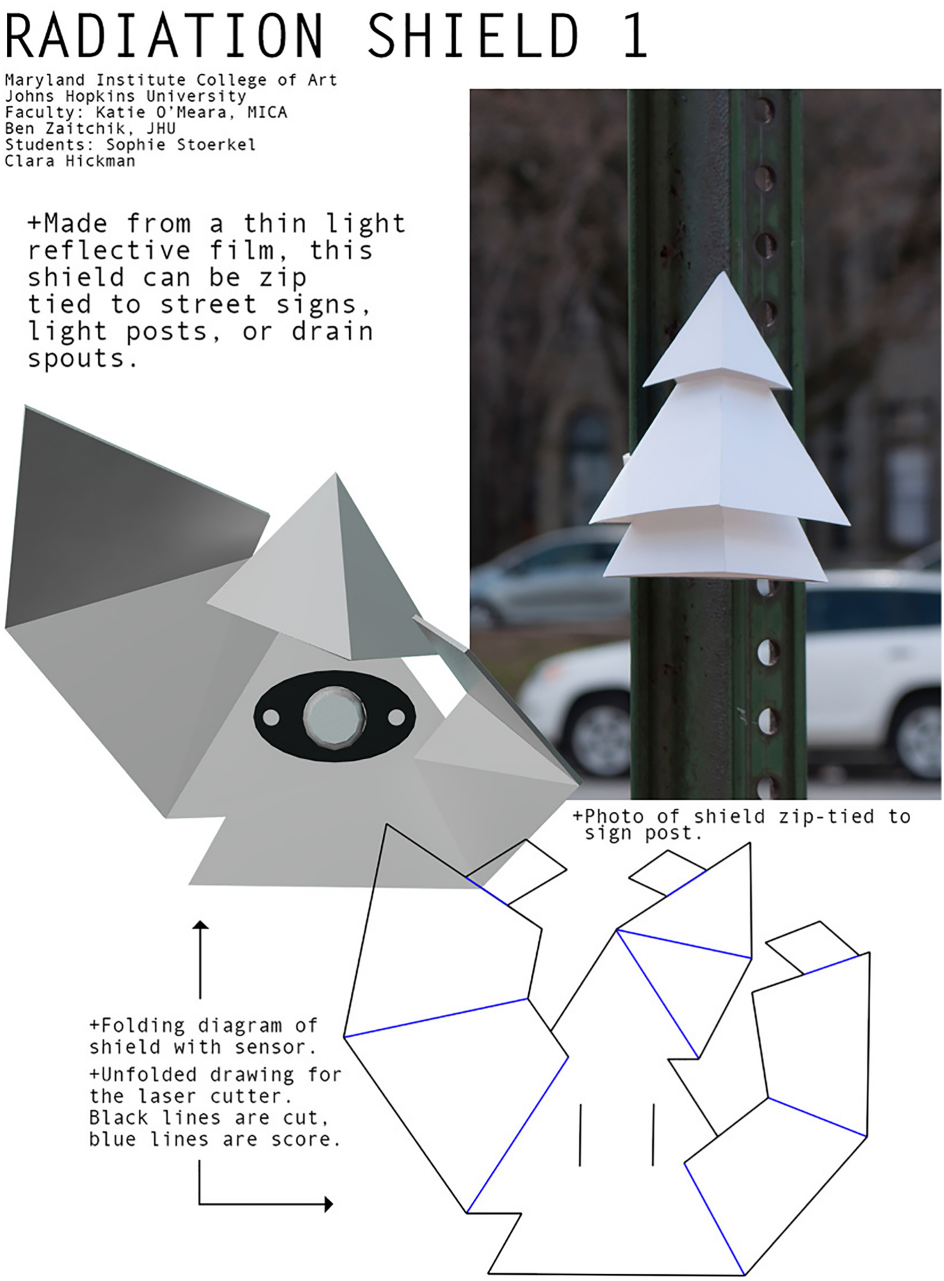
Schematic. A schematic of the iButton and radiation shield.

A network of Ibutton sensors (Fig 2) installed in three geospatially diverse Nairobi informal settlements (slums), Kibera, Mathare and Mukuru, provided the datasets used in this paper. Kibera is the largest informal settlements in Kenya and is situated southwest of Nairobi. Many houses in Kibera are densely built and accessed through narrow, unpaved paths. The primary construction materials comprise of galvanized iron sheet roofs, mud walls and concrete floors. There also exist gated concrete houses and flats with a mixture of asbestos and galvanized iron sheet roof tops built by the County Council of Nairobi. The second slum, Mathare, is located to the north east of Nairobi in a valley along Mathare River. The houses in Mathare are characterized by walls and roofs made of galvanized iron sheets. The houses are closely built leaving narrow access paths to people’s houses with little ventilation. A few sensors were also placed in the Mathare North neighborhood characterized by apartment buildings or flats, which are five to six stories high. The final slum, Mukuru, is located to the south east of Nairobi adjacent to the main industrial zone. The houses in this neighborhood are a mixture of a few high rise buildings and houses constructed from galvanized iron sheets (both walls and roofs), housing a significant number of people working within the surrounding industries.

**Fig 3:**
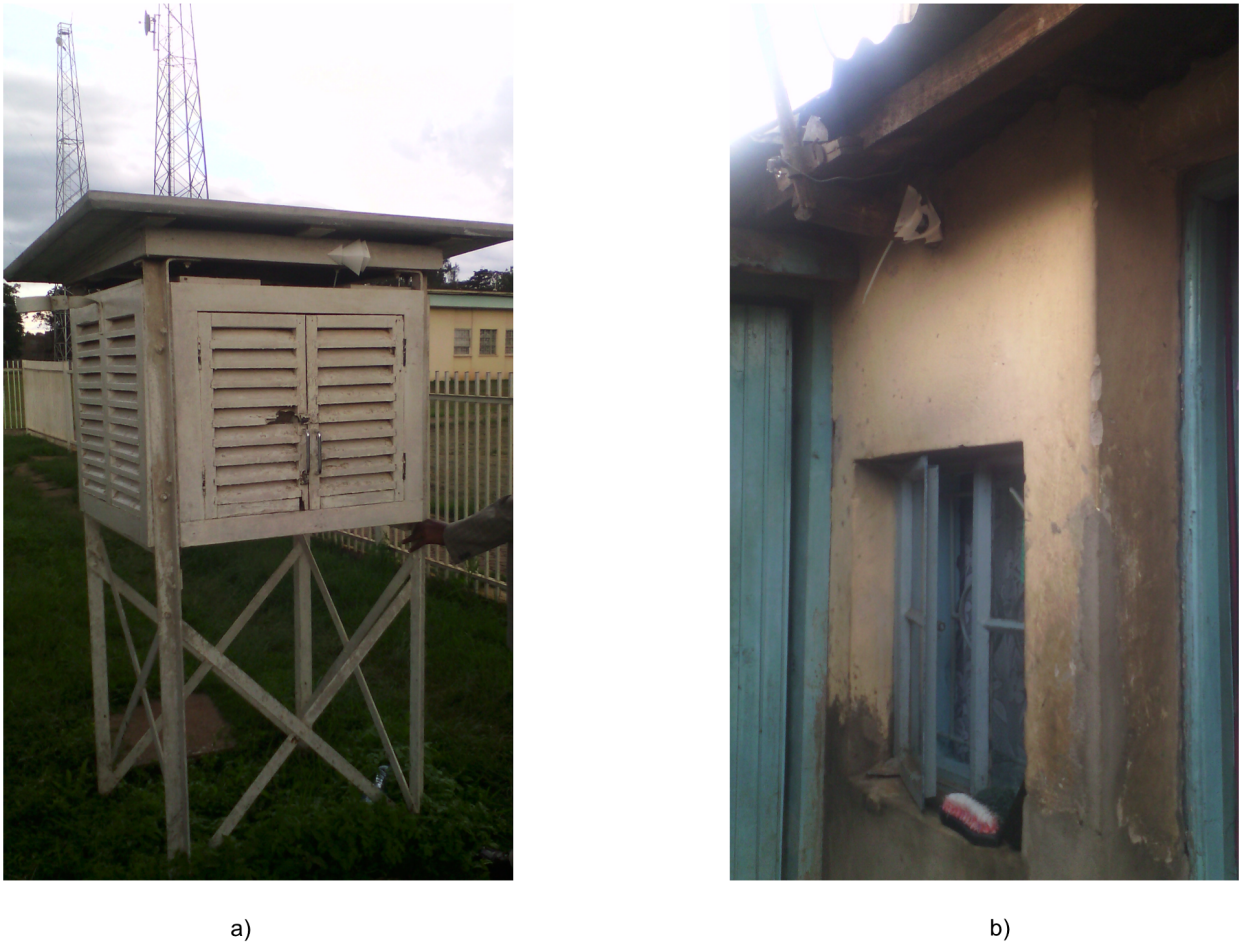
Study sites. The iButton thermometer/hygrometer installed *in situ*. a) shows the central monitoring site at the Kenya Meteorological Department headquarters and b) shows a typical household in the Kibera neighborhood.

We compare these sites to the Dagoretti weather station, situated at the Kenya Meteorological Department (KMD) headquarters (Fig 1) on a grassy, tree-lined campus (Fig 3a). The KMD headquarters is located inside the political boundaries of the Kibra constituency but approximately a kilometer away from the residential areas of Kibera. We also rely on a site at the University of Nairobi Chiromo Campus, a wooded and vegetation-filled campus that is fenced off from Nairobi Central Business District. We use four daily temperature variables from Dagoretti: 9am (local time) temperature, 3pm temperatures, minimum and maximum temperature. Minimum and maximum temperature are recorded from a min-max thermometer, an analogue instrument that captures the highest and lowest temperatures within a time period, in this case twenty-four hours, while the 9am and 3pm measurements are recorded from the station thermometer at 9 o’clock and 3 o’clock. We use Dagoretti’s daily minimum temperature from 1984 to 2014 to compute long-term climatological means for each day and Dagoretti’s sub-daily data (maximum and minimum temperature) from 2016 for temperature and relative humidity to validate the iButton data. The iButton thermometer/hygrometers, a product of Maxim Integrated, have an onboard data logger and are accurate to 0.5°*C* degrees. The sensors were housed in a custom, naturally aspirated radiation shield made of WhiteOptics White98 Reflector Film and the ensemble was attached with zip-ties to the eaves of houses, posts, and trees (Fig 2).

A network of 50 iButton sensors was installed in December 2015 and left to record through February 2016, at which time 43 remained (Fig 1). Sensors were installed facing North on or near private homes, schools, and non-profit organizations in parts of the Mukuru, Mathare, and Kibera neighborhoods. We also installed a sensor at the University of Nairobi Chiromo Campus observatory. iButtons were attached mostly to wooden posts (35 or 71%) and trees (6 or 12%) at a height of two to three meters. One sensor located at the Dagoretti station was installed on the metal frame of a Gill Screen at a height of 1.5 meters. Most sensors were installed in partial shade (22 or 45%) or full shade (23 or 47%); four were installed in full sun (8%). Five sensors were subsequently rejected for data quality concerns, including sensors attached to metal or in full sun, leaving 36 sensors for analysis.

Temperature and humidity observations are used to compute the heat index *HI*, a bi-quadratic function that estimates human heat exposure from relative humidity and temperature in Fahrenheit. Note that *T*°*F* = .9/5 ⋅ *T*°*C* + 32.

$$\begin{array}{c} HI=c_{1}+c_{2}T+c_{3}RH+c_{4}TRH+c_{5}T^{2}+c_{6}RH^{2}+c_{7}T^{2}RH+c_{8}TRH^{2}+c_{9}T^{2}RH^{2} \end{array}$$

where *c*_1_ = −42.37, *c*_2_ = 2.04901523, *c*_3_ = 10.14333127, *c*_4_ = −0.22475541, *c*_5_ = −0.00683783, *c*_6_ = −0.05481717, *c*_7_ = 0.00122874, *c*8 = 0.00085282, *c*_9_ = −0.00000199 [10]. Heat index is a unit-less quantity but can be understood as human-perceived temperature in degrees Fahrenheit.

### Data validation

iButton thermometers have been extensively validated in the literature [11], though a warm bias during daytime has been reported [12]. Using observations at three hour temporal resolution, we quantify the possible bias using three metrics. First, we calculate the percent bias (PBIAS) (1), the tendency of the iButton sensor to measure the average deviation of the iButton sensor reading *O* from the station record *S*. A value of zero is a perfect match; positive (negative) values indicate that the iButton overestimates (underestimates) the records compared to station instruments.

$$\begin{array}{c} PBIAS=\frac{\sum_{i=1}^{N}\left(S_{i}-O_{i}\right)}{\sum_{i=1}^{N}O_{i}}*100 \end{array}$$ (1)

Next, we calculate the Modified Index of Agreement or MIOA (2), which measures the degree of iButton error [13] between the iButton observations *O* and the station observations *S*. This metric varies between zero and one, with one indicating a perfect match and zero indicating no match.

$$\begin{array}{c} MOIA=1-\frac{\sum_{i=1}^{N}{\left(O_{i}-S_{i}\right)}^{j}}{\sum_{i=1}^{N}\left(\right|\left(S_{i}-\bar{O}\right)\left|+\right|\left(O_{i}-\bar{O}\right){\left|\right)}^{j}} \end{array}$$ (2)

Here, we use *j* = 2. We also calculate the mean difference in temperature and relative humidity, RH. We note that for the observation period of December 2, 2015 to February 20, 2016, data availability varies by meteorological parameter, as reported in Table 1.

**Table 1:** Instrument Evaluation Statistics. PBIAS, MIOA and mean difference for temperature and humidity measurements with respect to KMD instruments as well as the number of days the KMD data was available. The total observation period is 80 days.

time	PBIAS	MIOA	Mean Dif.	Days Available
9am	3.69	0.83	0.86	80
3pm	5.507	0.741	1.79	51
*T*_*min*_	0.19	0.877	0.16	79
*T*_*max*_	10.60	0.39	3.2	11
RH 9am	2.02	0.96	1.89	80
RH 3pm	-1.06	0.99	-0.71	51

**Fig 4:**
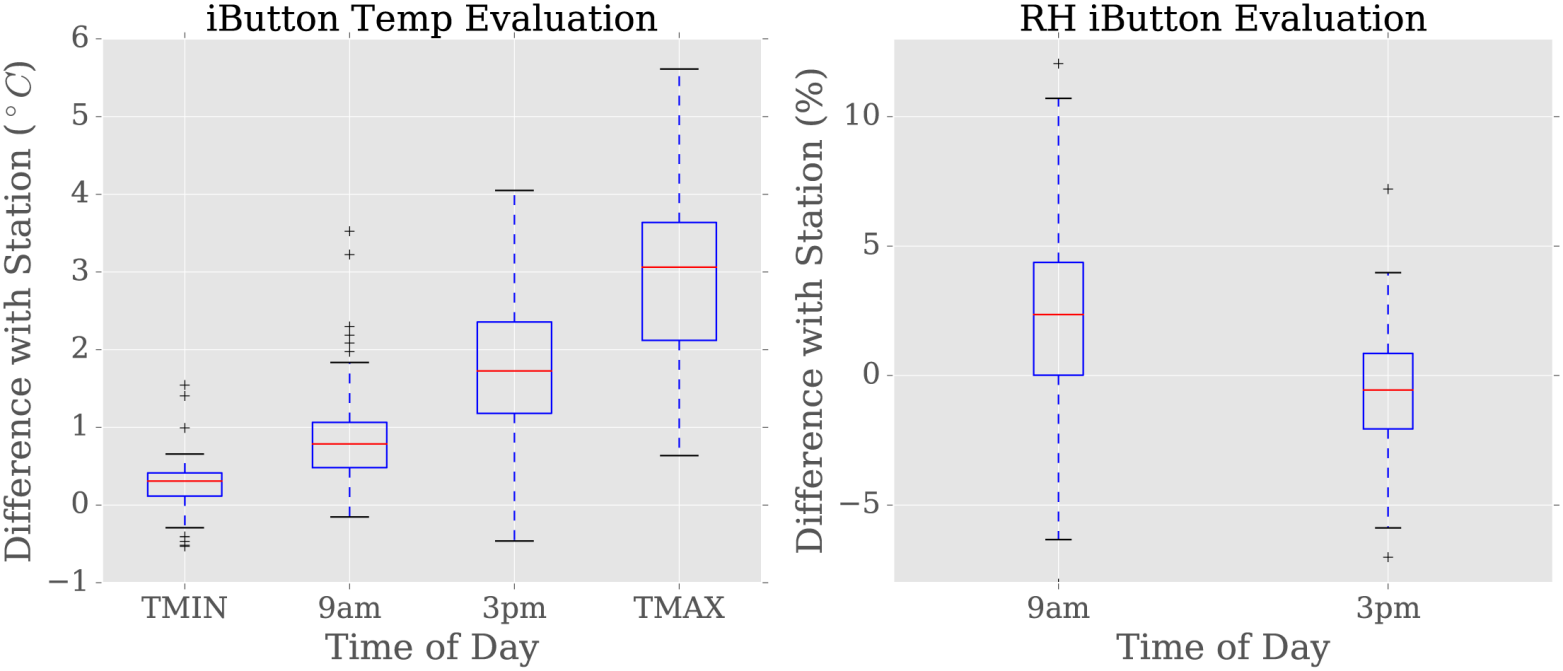
Instrument evaluation. Difference between an iButton co-located with the Dagoretti weather station and KMD station instruments. Note that hourly thermometer and min/max thermometer are different instruments.

Comparison with KMD instruments at Dagoretti—both an analogue thermometer as well as a min-max thermometer—confirms that thermometers agree well for morning measurements, but less well during the afternoon. Table 1 shows the results of our validation analysis for hourly data at 9am and 3pm local time, as well as minimum and maximum daily temperature, *T*_*min*_ and *T*_*max*_, respectively. The bias is highest for *T*_*max*_, but lowest for *T*_*min*_. Similarly, agreement is poorest for *T*_*max*_ and best for *T*_*min*_. Hourly agreement follows a similar pattern with bias lower and agreement higher for 9am than for 3pm, with a 3pm local time mean difference of 1.79°C (Fig 4). However, we note that the mean difference is larger for *T*_*max*_ than at 3pm (3.2 versus 1.79°C), despite the fact that maximum temperature occurs close to 3pm. Without overlapping data, it is difficult to determine the cause, but one possibility is that the different instruments have different responses and that perhaps the high bias for *T*_*max*_ may be overstated. Differences for *T*_*min*_ was not found to correlate with radiation, radiation lagged one day, or temperature. KMD data for *T*_*max*_ was unavailable for similar analysis.

Validation of iButton hygrometers has not been reported in the literature, though the manufacturer reports that the hygrometer has 5% precision. Our tests found good agreement with the station hygrometer (Table 1), with slight overestimation at 9am and slight underestimation bias at 3pm. This results in a low mean difference at 9 am (1.89%) and at 3 pm (-0.76%). As the MIOA values for humidity exceed those for temperature, we conclude that iButton hygrometer is not only a reasonable way to assess ambient humidity but furthermore, as MIOA is a normalized measure, more accurate throughout the day compared to the temperature reading.

We conclude that iButton data are a reasonably accurate representation of Nairobi’s nighttime thermal environment, especially for relative humidity, but perform less well in the afternoon. To account for this bias between iButton and Dagoretti instruments, this study uses the iButton sensor located in the same Gill screen as the Dagoretti thermometer as the ‘station temperature’. It is possible that this iButton may itself have a warm bias even compared to other iButton sensors because of its attachment to the shaded metal frame of the Gill screen. However, this error will underestimate station-neighborhood differences rather than overestimate.

### Satellite data

Satellite data from Landsat8 is used to calculate albedo, LST, and Normalized Digital Vegetation Index (NDVI), a proxy for vegetation abundance which detects photosynthetically active plants by looking at differences in their spectral reflectance of near-infrared light (NIR) and visible or red (VIS) spectrum light. The Landsat8 scene was taken on February 22, 2015 at 10am local time and downloaded from USGS’s EarthExplorer (https://earthexplorer.usgs.gov/). LST is calculated by inverting the Planck function and applying emissivity and atmospheric water vapor corrections as in [14]. Albedo is calculated as in [15] using a normalized form of [16]. NDVI is calculated as the normalized difference of the near infrared band *NIR* and the thermal infrared band *TIR*:

$$\begin{array}{c} NDVI=\frac{NIR-VIS}{NIR+VIS} \end{array}$$

NDVI functionally ranges from 0 to 1, and can be understood as the fraction of surface covered in vegetation.

### Statistics

We restrict the period of analysis to December 2, 2015 to February 20, 2016 in order to eliminate days when the network is only partially deployed. From this eighty day period, we calculate daily minimum and daily maximum temperature *T*_*min*_ and *T*_*max*_, which represent the maximum of hourly temperatures computed over the 24-hour period starting at midnight local time. Mean daily temperature or *T*_*mean*_ represents the mean of hourly temperature over the 24 hour period beginning at midnight. To understand spatial variability, we calculate the time-mean, denoted by $\bar{\cdot }$ for each sensor of minimum (maximum) daily temperature $\bar{T_{min}}$ ($\bar{T_{max}}$), which represents the average of minimum (maximum) daily temperatures at a given sensor for the 80 day period. We also examine the difference between the reference site at the Dagoretti weather stations and other temperatures for minimum and maximum daily temperature, Δ*T*_*min*_ and Δ*T*_*max*_.

To understand variability in time, we also compute averages with respect to space, denoted by <⋅> in a given neighborhood: <*T*_*Mathare*_>, <*T*_*Mukuru*_>, <*T*_*Kibera*_> as well as the standard deviation, *σ*_*Mukuru*_, etcetera. Additionally, to understand how 2015 relates to the historical record, we calculate an extreme temperature threshold of daily temperature using the Dagoretti station’s daily daily *T*_*min*_ and *T*_*max*_ from 1984-2014. To do so, we calculate the time-series of temperatures from each day of the year (e.g., January 1 1984, January 1 1985, *etc.*) and calculate the 95^th^ percentile, resulting in a daily time-series of 95th percentiles. We apply a monthly (30 day) rolling mean to smooth this. This threshold makes use of KMD instruments; in order to compare with our own instruments, we adjust it by the mean instrument bias in *T*_*min*_ and *T*_*max*_ (Table 1).

## Results

### Temperature

**Fig 5:**
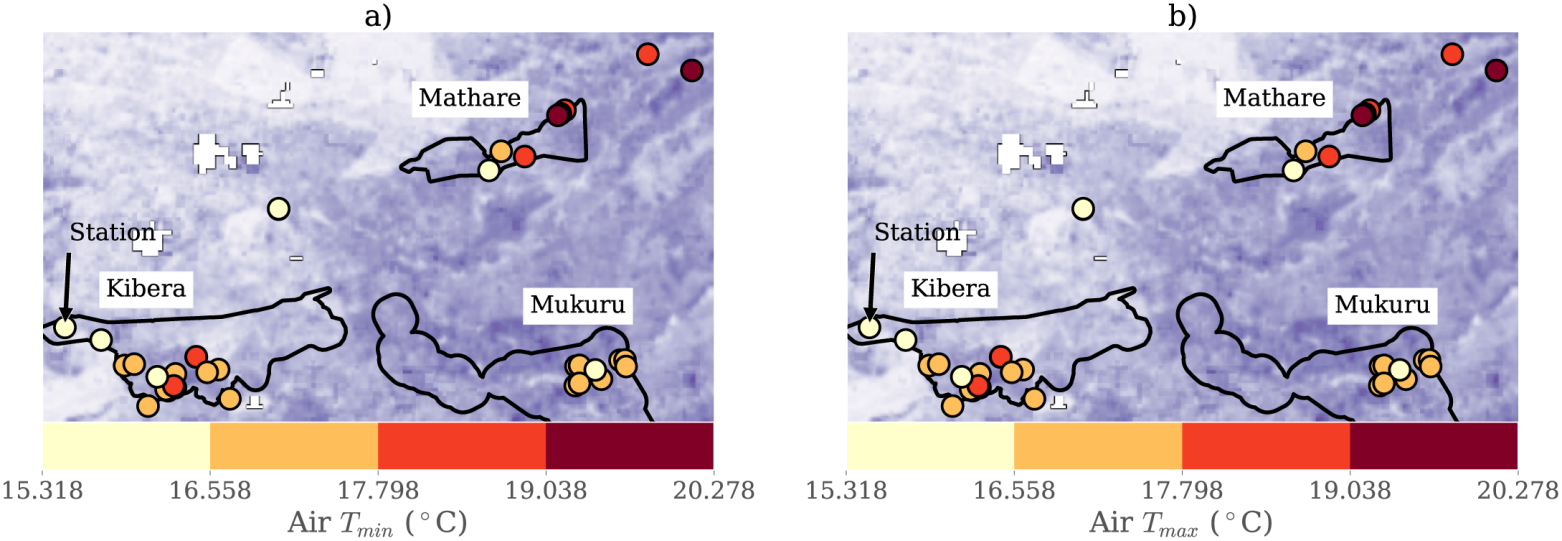
Mean temperature by location. Mean temperature for a) minimum daily temperature and b) maximum daily temperature for each sensor for summer 2015-2016 in Nairobi.

Measurements show that air temperature in Nairobi varies geographically, and that on average, sensors located in informal settlements are warmer than the sensor located at the reference site at the Dagoretti weather station (hereafter, “station”). Fig 5a and 5b maps $\bar{T_{min}}$ and $\bar{T_{max}}$ for each sensor by location, and demonstrates that despite its physical proximity to informal settlements in Kibera, the station is cooler than most sites for both *T*_*min*_ and *T*_*max*_. Minimum and maximum daily temperature occur at approximately 6am and 3pm local time. Monitors have a mean difference of Δ*T*_*min*_ = 1.83°*C* and Δ*T*_*max*_ = 3.1°*C* from the station.

**Fig 6:**
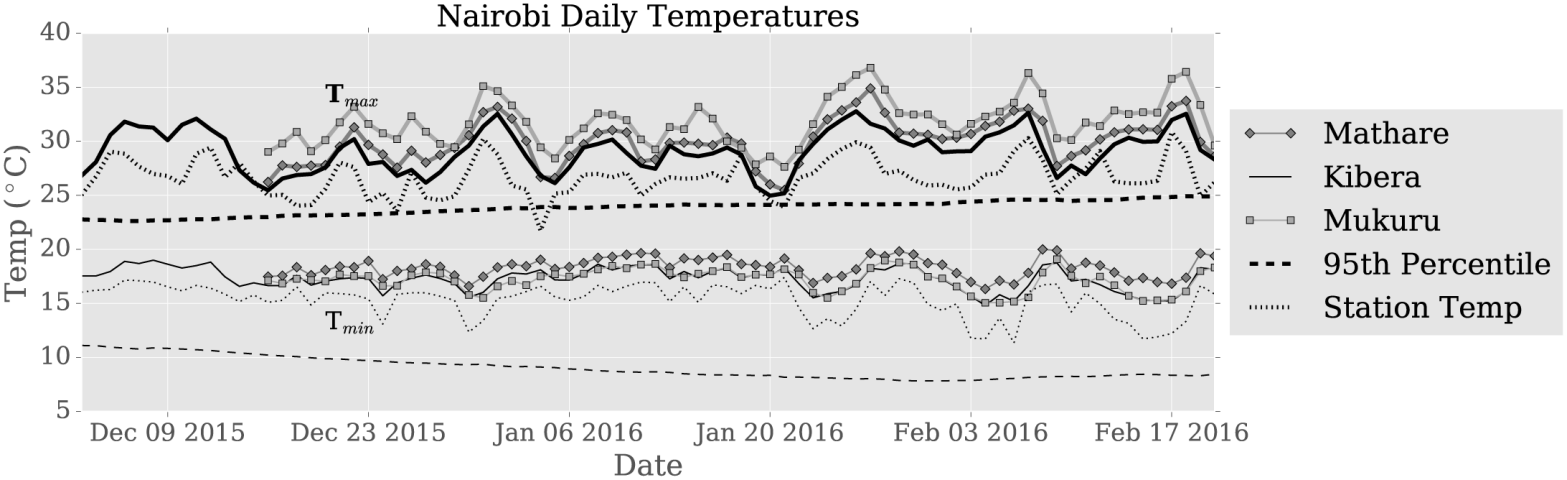
Temperature by time. Minimum and maximum daily temperature for each the ensemble average of iButtons in each neighborhood (solid lines) and the iButton located at the central monitoring station (dotted lines). The 95^th^ percentile of 1984-2014 temperature (dashed black) for minimum and maximum temperature is calculated from the Dagoretti station and adjusted by the mean iButton-Dagoretti instrument difference to correct for instrumentation bias.

The spatial variability of temperature is consistent throughout the summer, meaning that relative to the sample mean, hot sensors are consistently warmer, and cool sensors are consistently cooler, though day-to-day variability exists particularly for sensors near the sample average. This can be seen in Fig 6, which shows the time-series of daily temperature <*T*_*min*_> (top lines) and <*T*_*max*_> (bottom lines) at the reference site as well as for each neighborhood’s average. Thus, we conclude that the differences observed with the station are robust in time and space.

Summer 2016 was the hottest in 30 years in Nairobi and temperatures exceeded a number of climatological heat thresholds (Fig 6), including minimum daily temperatures being more than 2°C above the 1967-1999 climatological mean minimum daily temperature. The heat persisted throughout the summer, and day-to-day variability was low, only 1.0°C, as measured by the standard deviation of the timeseries of daily *T*_*mean*_. Both *T*_*max*_ and *T*_*min*_ exceed the 95th percentile of climatological temperature (after adjustment for instrument bias) on most days: at the station, all 80 days exceed the *T*_*min*_ threshold and 76/80 or 95% exceed the *T*_*max*_ threshold. In the neighborhoods, all days exceed the threshold. Furthermore, varying the reference period used to calculate the 95^th^ percentile to 2000-2015 did not affect this result significantly and only resulted in a few additional days at the station not exceeding the threshold.

**Fig 7:**
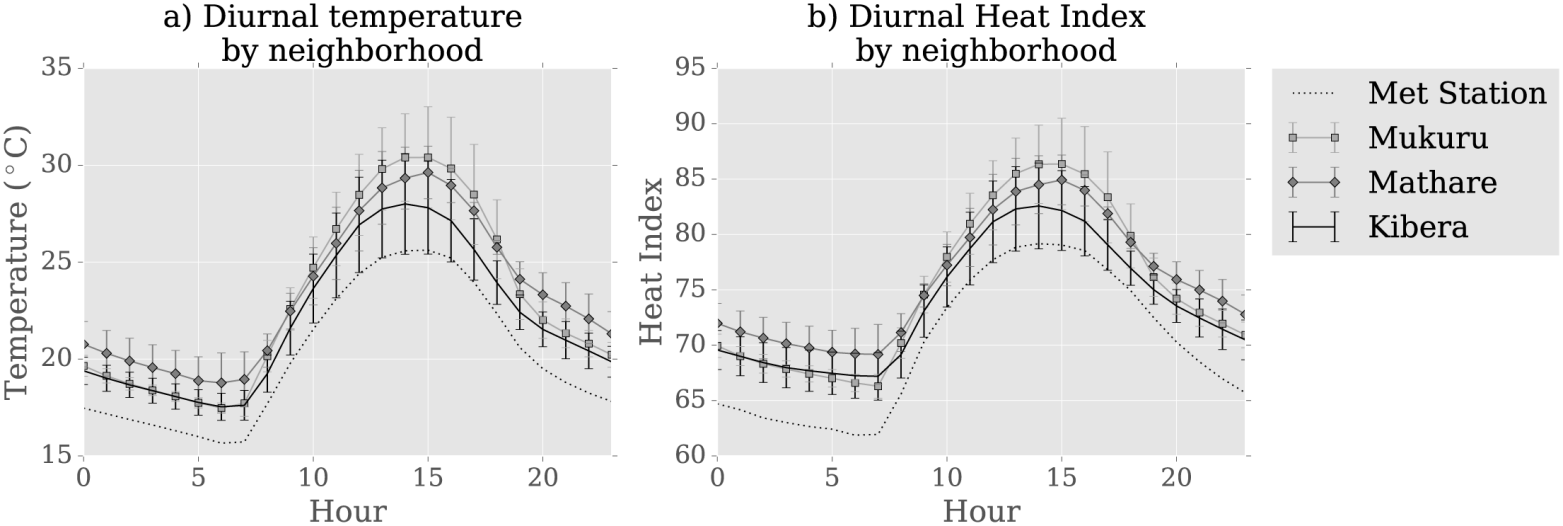
Mean temperature by hour. Diurnal cycle of a) mean hourly temperature in each neighborhood and b) mean heat index by neighborhood. Error bars show the sensor-to-sensor standard deviation, representing spatial variability.

Urban-rural differences are thought to be largest at night [3]. In Fig 7, we examine mean temperature by hour in each neighborhood and see that there are nighttime differences with the Dagoretti weather station, but these differences persist throughout the day and are in fact largest during daytime hours. The Mathare neighborhood is hottest at night, with <Δ*T*_*min*_ > = 3.2°*C*, whereas the Mukuru neighborhood is hottest during the day, with <Δ*T*_*max*_ > = 4.8°*C*. Error bars in Fig 7 represent spatial variability in each neighborhood, calculated by taking the standard deviation of same time-of-day measurements averaged across all days. Spatial variability is larger during the day than at night, largest during the afternoon, and also varies somewhat by neighborhood. The daytime spatial variability is highest in Kibera and Mukuru, peaking at *σ*_*Kibera*_ = 2.6°*C* and *σ*_*Mukuru*_ = 2.6°*C*, while maximum variability in Mathare is slightly lower, *σ*_*Mathare*_ = 2.1°C.

### Humidity

Humidity can exacerbate or moderate the impact that high temperature has on heat exposure. We examine the effect of humidity on thermal comfort by using the heat index, and find that while temperature is higher in informal settlements than relative humidity is lower. These geographic differences in humidity, however, are not enough to offset the impact that temperature has on the heat index in informal settlements. The net result is that the heat index is elevated in informal settlements than at the central monitoring site. This is shown in Fig 7b, which shows mean hourly heat index by neighborhood and the spatial variability in heat index. These curves look similar to those calculated for temperature in Fig 7a, indicating that the heat index difference between sites is similar to the site-to-site temperature differences. Data was not available to compute other heat exposure metrics such as apparent temperature.

### Proposed explanatory mechanism

**Fig 8:**
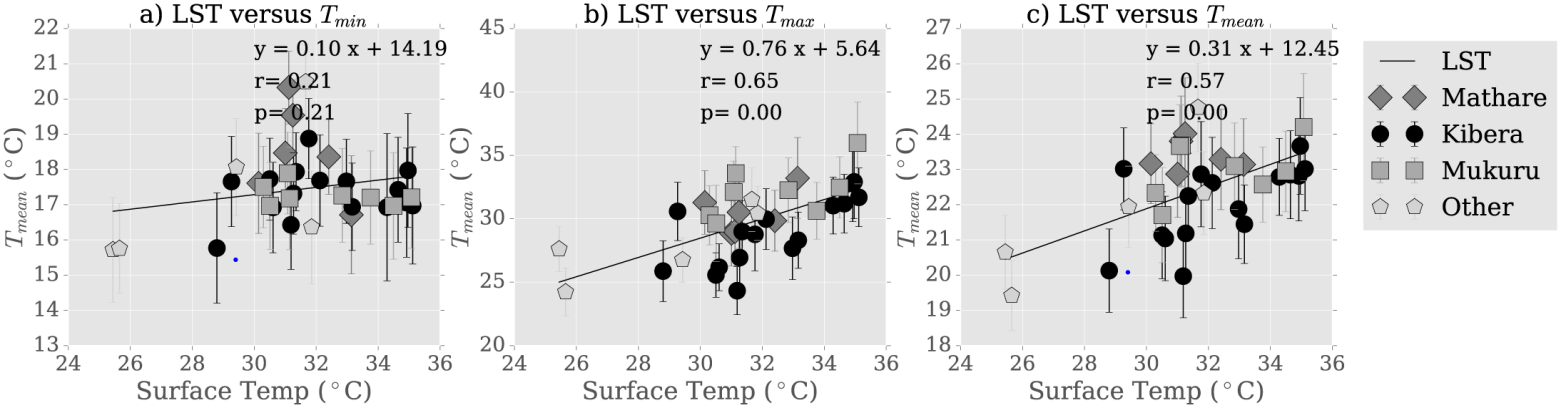
Relationship with LST. Relationship between LST and a) *T*_*min*_, b) *T*_*max*_, and c) *T*_*mean*_.

The observed geographic variability can be explained in part by the variations in surface properties. Urban heat excess is often related to surface properties, though it can also be caused by anthropogenic emissions of heat and pollutants into the atmosphere. Our measurements find evidence of land-atmosphere coupling in Nairobi informal settlements, indicating that surface properties are responsible for much of the observed urban heating. This can be seen in the strong correlation between LST and mean daily air temperature (Fig 8) (*r* = 0.61, *p* = 0.00). The correlation is strongest for mean daily temperature, and weakest for minimum daily temperature, suggesting that surface properties control heating and, to a lesser extent, cooling, and that optimal heat mitigation strategies must reduce daytime heating in addition to increasing nighttime cooling.

The urban surface energy budget can be written in terms of surface properties:

$$\begin{array}{c} R_{net}=H+L+G \end{array}$$

where *R*_*net*_ is the net radiative budget, *H* is sensible or dry heat, *L* is the latent heat of evaporation or condensation from evapotranspiration, and *G* is ground flux of excess heat [17]. While we have not measured these terms directly, we can remotely sense proxies for these surface variables using satellite data, namely elevation, vegetation abundance, and surface reflectivity or albedo. A multiple linear regression shows that much of the spatial variability in mean daily air temperature (66%) can be explained by these surface factors:

$$\begin{array}{c} <T_{mean}>=-0.7green-0.007elevation-7.8albedo-4.5NDVI+35.8 \end{array}$$

**Fig 9:**
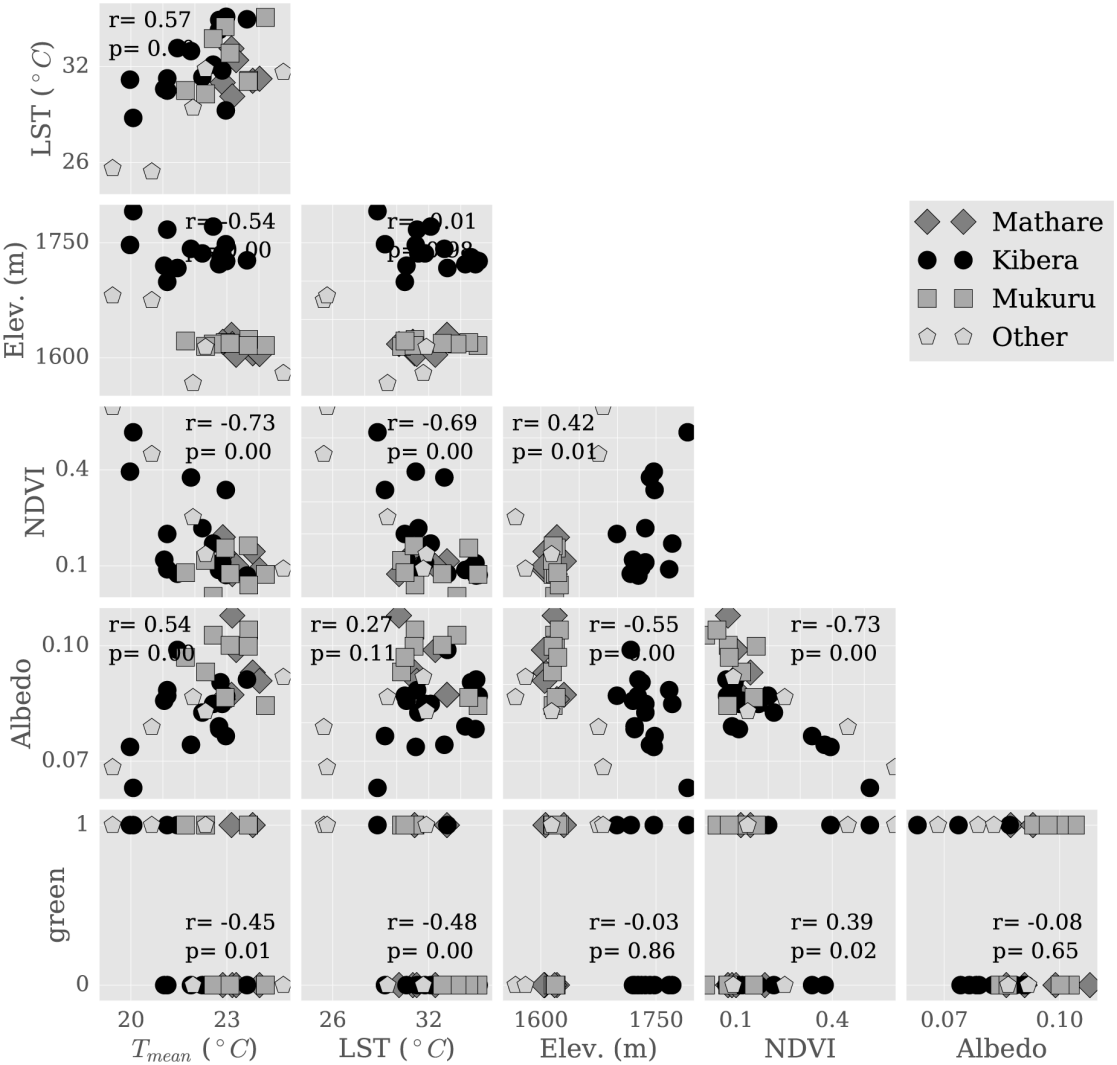
Relationship of temperature and surface variables with each other. Scatter plots showing the relationship between mean daily temperature *T*_*mean*_, land surface temperature (LST), elevation, NDVI, albedo, and *green*. NDVI, albedo, and *green* are unit-less quantities ranging from 0 to 1. Top left shows *T*_*mean*_ versus LST, second row shows (from left to right) *T*_*mean*_ versus elevation and LST versus elevation, etcetera.

Except for *albedo*, all covariates are statistically significant at 95%. Here, *green* represents the presence of vegetation near the sensor as measured *in situ* during sensor deployment and is an indicator variable equal to 0 (no vegetation present) or 1 (site dominated by vegetation). Its coefficient indicates that moving to a vegetated location results in a mean cooling of approximately 0.7°*C*. This variable is distinct from NDVI, a continuous variable which ranges from 0-0.6 in our study locations (see Fig 9, 3rd and bottom row) and is capturing greenness seen from above rather than ground-level plants. NDVI represents green intensity rather than a percent coverage, and so cannot be directly related to *green* without further ground truthing. The NDVI coefficient indicates that moving to entirely vegetated locations will result in a cooling of up to 4.5°*C* if an NDVI of 1 can be achieved, though NDVI rarely attains this value, so a more likely maximum cooling value for this area is 2.7°*C*. The discrepancy with *green* could be interpreted as a function of scale, but more likely points to a variety of vegetation characteristics that affect temperature.

The regression result suggests that vegetation plays a regulating role on urban climate in Nairobi. *T*_*mean*_ correlated significantly with NDVI (Fig 9, 1st column, 3rd row, *r* = −0.73, *p* = 0.00), though we note that *T*_*max*_ exhibited stronger correlations with NDVI than with *T*_*min*_ (*r* = −0.67 versus *r* = −0.47). As vegetation affects evapotranspiration and thus latent heat release, this is consistent with the hypothesis that vegetation is effective at reducing daytime heating and that latent heating plays an important control on Nairobi climate. This result does, however, carry the caveat that vegetation influences instrument sun exposure during the day, possibly affecting differences in *T*_*max*_ site-to-site.

Furthermore, a combination of these surface properties may help explain thermal differences between neighborhoods. While the vegetation fraction is overall low, we note that Mukuru, the neighborhood hottest during the day, has the lowest measured vegetative fraction (*NDVI*_*Mukuru*_ = 0.09), half of that in Kibera: *NDVI*_*Kibera*_ = 0.189 (*NDVI*_*Mathare*_ = 0.12) (Fig 9, third row). Another factor is elevation, which will affect radiative efficiency and possibly wind speed and thus the nighttime cooling rate. Kibera is the highest elevation neighborhood: $\bar{h_{Mathare}}=1614\text{m}$, compared to $\bar{h_{Mukuru}}=1618\text{m}$ and $\bar{h_{Kibera}}=1741\text{m}$, further explaining why it remains cooler during the day (see Fig 9, second row, first column). Mathare has the lowest median elevation (*overlineh*_*Mathare*_ = 1612), further contributing to nighttime heat. This analysis omits some properties, notably surface geometry and the sky view factor, which can play important roles in thermal regulation. Nevertheless, physically consistent relationships between temperature and elevation, albedo and vegetation suggest that these surface properties are important to temperature variability within and around informal settlements.

### Impact on health

**Fig 10:**
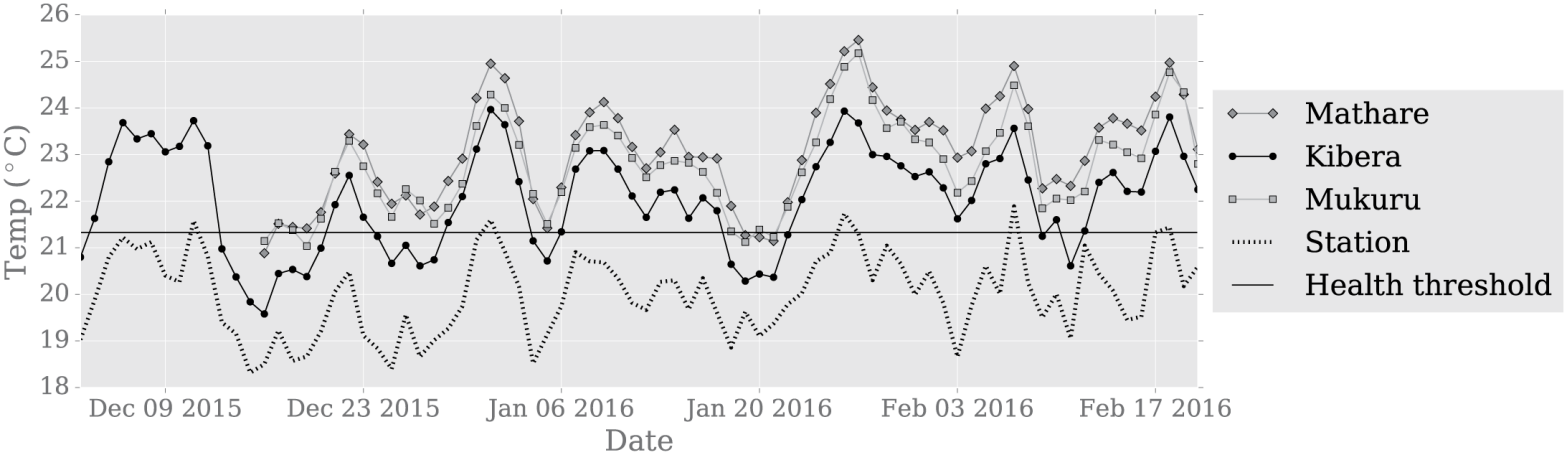
Temperature and health thresholds. Mean daily temperature for each the ensemble average of iButtons in each neighborhood (solid lines) and the iButton located at the central monitoring station (dotted line). The heat threshold is the 20°*C* threshold at which statistically significant increases in mortality are observed, measured at the Moi weather station, in [9], adjusted by the mean 9am and 3pm iButton-Dagoretti instrument difference to correct for mean instrumentation bias. Mean temperatures are computed as the average of hourly temperatures.

The warmer conditions seen in summer 2015/2016 increase the likelihood of negative health impacts. Epidemiologists have found that extremely high heat increases mortality and morbidity, where an “extreme” is frequently defined as when temperature exceeds the 95^th^ percentile of the long term temperature record (e.g., [18]). While more accurate long term data within neighborhoods would be needed to determine what the 95th percentile of local temperature is, negative health effects have been seen in children and the elderly in Kibera at temperature thresholds as low as the 75^th^ percentile [9], found to be 20°*C*, as measured at the Moi Airbase weather station in Nairobi. That study found that mortality in age groups 0-4 and 50+ increased by 1% for every 1°C increase in mean daily temperature above 20°*C*. Temperatures met or exceeded this threshold on 5 days at the station during the measurement period, accounting for instrument differences in hourly temperature (Fig 10). On these days, temperatures in the neighborhood exceeded the central monitoring station by several degrees. This suggests that increases in mortality are possible on the hottest days, but that also that the health threshold in [9] is probably representative of higher local temperatures. We note that this relationship was calculated using another station which may exhibit different biases than the KMD instruments for which we have data, but we did find that the station temperature record used in this paper was consistent with the mean, max, and threshold data reported from the Moi station in [9].

The thermal differences observed between the Dagoretti weather station and neighborhoods potentially put the examined neighborhoods at greater risk for heat-related illness. Kenya does not have a national heat alert system, but generally, national meteorological agencies issue heat alerts when temperature or the heat index exceeds thresholds, either absolute or relative, allowing public health and disaster management professionals to respond. If station temperature remains below a given threshold when neighborhood temperatures exceed the threshold, disaster management professionals may fail to provide relief because they do not have accurate information on conditions in vulnerable communities. We find that this is a concern for Nairobi as informal settlements are hotter than the threshold temperature so consistently, but the station is not. The station temperature exceeded the [9] threshold for only 6% of days (5/80), meaning that heat alerts relying on this threshold would have failed to issue a heat alert on most days (Fig 10), assuming that. We note, however, different thresholds may have had more success; the use of a daily heat threshold calculated for each day’s temperature (e.g., as in Fig 6) would have had higher accuracy.

**Fig 11:**
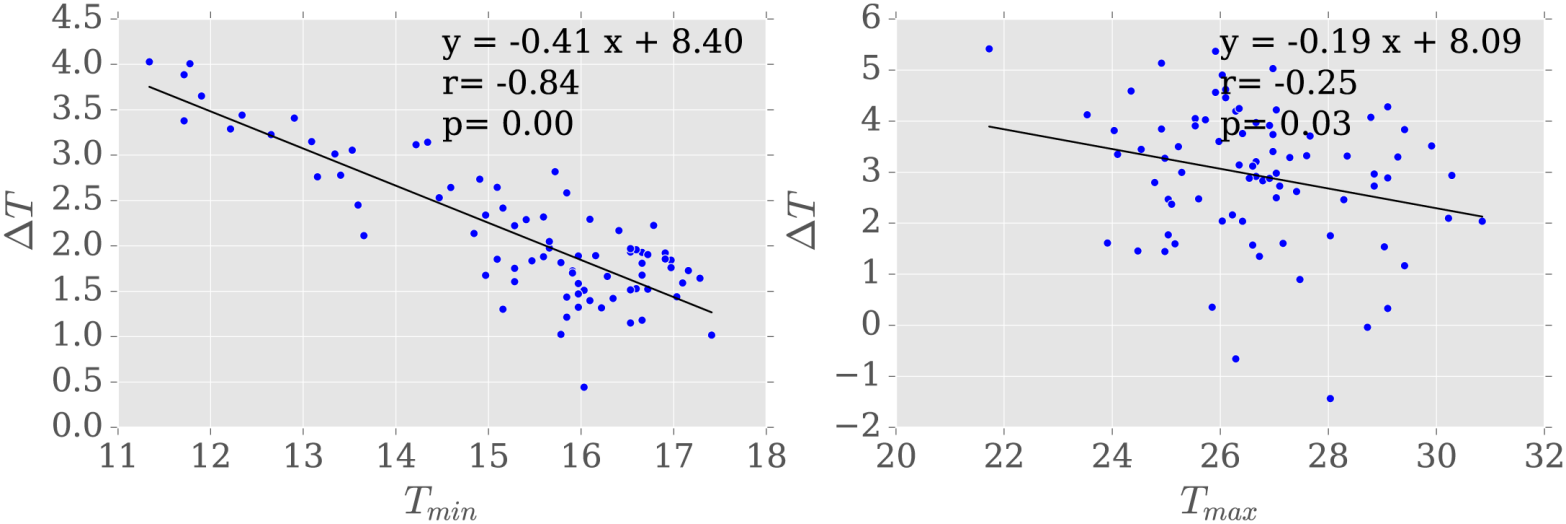
Temperature versus temperature difference. Relationship between (a) *T*_*min*_ and (b) *T*_*max*_ and Δ*T*, the mean difference between Nairobi neighborhood and the central monitoring station. Black line shows the linear regression line.

As it grows hotter, daytime sensor-to-sensor variability increases, as measured by the spatial standard deviation of sensors temperatures (*r* = 0.4, *p* < 0.00). However, as minimum daily temperatures increase, nighttime sensor-to-sensor variability (standard deviation) decreases. Moreover, as temperatures rise, the mean difference between sensors and the station 〈Δ*T*〉 decreases (Fig 11). This is particularly true for nighttime temperatures (*r* = −0.84, *p* < 0.05), but a significant relationship is also found between daytime temperatures and the difference with the station (*r* = −0.25, *p* = 0.03). We quantify this difference using a linear regression of the form Δ*T* = *mT*_*station*_ + *b*, where *m* denotes the sensitivity of Δ*T* to *T*_*station*_ (black line, Fig 11). For *T*_*min*_, *m* = −0.41°/°C and for *T*_*max*_, *m* = −0.19°/°C, suggesting that as temperature increases by a degree, station differences decrease by 0.2–0.4°C. Ultimately, this effect is not enough to reverse the sign of Δ*T*, which remains positive for all but 3 days.

## Conclusions

During the hottest summer on record in Nairobi, temperatures measured within 3 informal settlement neighborhoods— Kibera, Mathare, and Mukuru— regularly exceed temperatures at the central, non-slum monitoring station by several degrees or more. These differences persist throughout the day and night. The spatial patterns observed in temperature are consistent over the measurement period—that is to say, hot stations remain hotter, and cool stations remain cooler. This spatial variability increases during periods of extreme heat, though the mean differences with the station tend to be smaller as it warms. This is particularly true for maximum daily temperature, though the station remains hotter than the spatially averaged temperature for the entire measurement period. We can connect much (66%) of the site-to-site variability in mean temperature to surface properties. In particular, the presence of vegetation (measured remotely and *in situ*) is a significant predictor of cooler mean temperatures.

These measurements suggest that Dagoretti weather station underestimates the heat exposure that is experienced by residents of informal settlements. Temperatures measured within neighborhoods are within the range of temperatures that have previously been associated with negative health outcomes for children and elderly populations [9]. Ultimately, our results suggest that some of Nairobi’s urban heating may be mitigated through improved urban design and increased greenery, though more data is needed to assess this.

Several questions remain outstanding for understanding heatwaves, the heat island, and their interaction in Nairobi and similar cities. Future studies should consider a broader range of neighborhoods to understand how heat and heat exposure may affect all residents. Finally, we note that we took measurements in an extraordinarily hot year. As temperatures rise and hot summers like 2015 become more common, a fuller understanding of how the urban heat island interacts with heatwaves will become more important. Such an understanding may aid in the development of more targeted interventions to reduce the effects of heat exposure now and in a warmer climate.
